# Trends in In-Hospital Cardiopulmonary Resuscitation from 2010 through 2019: A Nationwide Cohort Study in South Korea

**DOI:** 10.3390/jpm12030377

**Published:** 2022-03-01

**Authors:** Tak Kyu Oh, You Hwan Jo, In-Ae Song

**Affiliations:** 1 Department of Anesthesiology and Pain Medicine, Seoul National University Bundang Hospital, Seongnam 13620, Korea; airohtak@hotmail.com; 2 Department of Anesthesiology and Pain Medicine, College of Medicine, Seoul National University, Seoul 04551, Korea; 3 Department of Emergency Medicine, Seoul National University Bundang Hospital, Seongnam 13620, Korea; drakejo@snubh.org

**Keywords:** cardiopulmonary resuscitation, critical care, heart arrest, hospitals, resuscitation

## Abstract

We aimed to examine recent trends in in-hospital cardiopulmonary resuscitation in South Korea from 2010 to 2019. A population-based sample of all adult patients who experienced in-hospital cardiopulmonary resuscitation between 1 January 2010 and 31 December 2019, was included. In all, 298,676 patients who received in-hospital cardiopulmonary resuscitation were included in the survival analysis. In 2010, 60.7 per 100,000 adults experienced in-hospital cardiopulmonary resuscitation. A similar rate was observed until 2015. The rate increased to 83.5 per 100,000 adults in 2016 and gradually increased to 92.1 per 100,000 adults in 2019. Among all patients, 78,783 (26.2%) were discharged alive after in-hospital cardiopulmonary resuscitation. The 6-month and 1-year survival rates were 9.8% and 8.7%, respectively. In 2010, the mean total cost of hospitalization was USD 5822.80 (United States Dollar) (standard deviation; SD: USD 7493.4), which increased to USD 7886.20 (SD: USD 13,071.6) in 2019. The rate of in-hospital cardiopulmonary resuscitation and cost of care have significantly increased since 2010, while the 6-month and 1-year rates of survival post in-hospital resuscitation remain low.

## 1. Introduction

In-hospital cardiac arrest (IHCA) is an acute and critical event that can cause death in any hospitalized patient [1]. The occurrence of IHCA is common in United States hospitals, with a survival rate as low as approximately 20% [2]. In South Korea, the prevalence of IHCA was 2.46 per 1000 admissions in 2009, according to the National Representative Patient Sample [3].

Cardiopulmonary resuscitation (CPR) is a life-saving procedure that is required for patients who have a cardiac arrest event, and in-hospital CPR (ICPR) should be performed for patients with IHCA [4]. In the United States, the incidence of ICPR has increased, and the overall survival rate was 30.4% from 2007 to 2012 among non-elderly (18–64 years) patients [5]. Another epidemiologic study reported that, in the United States, 18.3% of elderly patients (≥65 years) survived to discharge after ICPR. In South Korea, according to the National Representative Patient Sample database, there were 5919 ICPR cases from 2003 to 2013, and the live discharge rate was 11.7% [6]. However, there have been no detailed reports on recent trends regarding prevalence, mortality, factors associated with hospital mortality in South Korea, clinical characteristics, or associated costs for patients who receive ICPR.

Therefore, we aimed to examine recent trends in ICPR in South Korea from 2010 to 2019 using the National Health Insurance Database. Given the increase in the aging population in South Korea, the frequency of ICPR due to IHCA is likely to increase. However, hospital mortality rates after ICPR may have improved due to advances in critical care medicine.

## 2. Materials and Methods

### 2.1. Study Design, Setting, and Ethical Concerns

For this nationwide, population-based cohort study, we followed the “Strengthening of the Reporting of Observational Studies in Epidemiology” guidelines [7]. The study protocol was approved by the institutional review board (IRB; X-2011-651-901), and the National Health Insurance Service (NHIS) permitted data sharing after approval of the study protocol (NHIS-2021-1-266). The requirement for informed consent was waived by the IRB because anonymized data were used in this study.

### 2.2. Data Source

The NHIS database was used for this study. As the sole public insurance database system in South Korea, it contains information regarding all disease diagnoses and medication prescriptions and/or procedures. These registrations enable patients to receive financial support from the government for treatment expenses. The International Statistical Classification of Disease and Related Health Problems, 10th Revision (ICD-10) codes were used to diagnose diseases.

### 2.3. Study Population

We initially screened patients who underwent CPR between 1 January 2010 and 31 December 2019. Next, we excluded cases of CPR for out-of-hospital cardiac arrest, and all ICPR cases were selected for this study. For any patient, all cases of ICPR in a day were counted as one ICPR case. For example, if a patient received ICPR four times on a certain day during the study period, it was considered as one ICPR case. If a patient received ICPR two or more times on different days during the study period, only the first ICPR case at the earliest date was included in this study. Pediatric patients were excluded from the analysis. The accurate death dates for all patients included in the study population were extracted and collected until 30 April 2021.

### 2.4. Collected Information

Age and sex were collected as the physical information. To reflect the socioeconomic statuses of the patients included in the study population, employment status, residence, and household income level at ICPR were collected. Self-employed patients were not considered as employed, and residence data, such as residency in urban areas (Seoul and other metropolitan cities) or rural areas, were collected at hospital admission based on ZIP codes. The NHIS collects information regarding household income levels to determine insurance premiums for patients, and the data were divided into four groups using the quartile ratio.

The main diagnoses at ICPR were collected and divided into four groups, according to ICD-10 codes: cardiovascular disease (I00–I99), respiratory disease (J00–J99), cancer (C00–D49), and others. The main diagnosis at ICPR was determined by NHIS after hospital discharge or death as the disease that required the greatest treatment or examination during the patient’s hospitalization. The admitting departments at ICPR were identified and classified into internal medicine (IM) and non-IM groups. The duration of ICPR was classified into one of five groups: <15 min, 15–30 min, 30–45 min, 45–60 min, and >60 min. The total length of hospital stay (day), and total hospitalization cost (United States Dollar, USD), were collected. The hospitals where the ICPRs were performed during the study period were classified into three groups: tertiary general hospital, general hospital, and other hospital. In addition, hospitals were divided into two groups, according to the total number of hospital beds, including those in intensive care units: <1000 beds and ≥1000 beds. The results of treatment at ICPR were collected and classified into four groups: discharge and same hospital follow-up, transfer to a long-term facility care center, death during hospitalization after ICPR, discharge, and other outpatient clinic follow-up. Lastly, to reflect the comorbid status of all patients, the Charlson comorbidity index (CCI) score was calculated using ICD-10 codes in the NHIS database, which were registered no more than 1 year before ICPR, as shown in Table S1.

### 2.5. Study Outcomes (Trend of ICPR in South Korea)

First, we examined the prevalence of ICPR between 2010 and 2019. The prevalence of ICPR was calculated as the total number of annual ICPR cases divided by the total adult population in that year. The total population was obtained from the Statistics Korea database (http://kostat.go.kr/portal/eng/index.action, accessed on 7 September 2021). Second, the in-hospital, 6-month, and 1-year mortality rates from 2010 to 2019 were examined. Third, the trend in total costs of hospitalization at ICPR was examined. Fourth, the main diagnosis and total duration of ICPR were examined. Fifth, the factors associated with live discharge after ICPR were examined.

### 2.6. Statistical Analysis

The clinicopathological characteristics of the study population were presented as mean values with standard deviation (SD) for continuous variables and numbers with percentages for categorical variables. We constructed a multivariable logistic regression model for the live discharge rate for patients who experienced ICPR. All covariates were included in the multivariable model, and the results are presented as adjusted odds ratios (aORs) with 95% confidence intervals (CIs). The goodness-of-fit in the multivariable model was confirmed using the Hosmer–Lemeshow test, and there was no multicollinearity issue between variables with criteria of variance inflation factors <2.0. All statistical analyses were performed using SPSS software (IBM SPSS Statistics for Windows, Version 25.0, IBM Corp., Armonk, NY, USA), and statistical significance was set at *p* < 0.05.

## 3. Results

### 3.1. Study Population for Survival Analysis

Figure S1 shows the study population selection process. From 1 January 2010 to 31 December 2019, there were a total of 478,836 CPR cases in South Korea. After the exclusion of 140,046 cases of CPR due to out-of-hospital cardiac arrest, 338,970 ICPR cases were initially screened. Next, 31,122 cases in which a patient received ICPR on two or more days during the study period and 8992 pediatric cases (patients under 18 years of age) were excluded from the final analysis. Finally, 298,676 patients were included in the survival analysis. Among them, 78,783 (26.2%) were discharged alive after ICPR, and the 6-month and 1-year survival rates were 9.8% (29,303/298,676) and 8.7% (25,850/298,676), respectively. The clinicopathological characteristics of the study population are shown in Table 1. The mean age was 70.0 years old (SD: 15.2 years old), and the proportion of male patients was 60.4% (180,389/298,676).

### 3.2. Trends of ICPR

**Figure 1:**
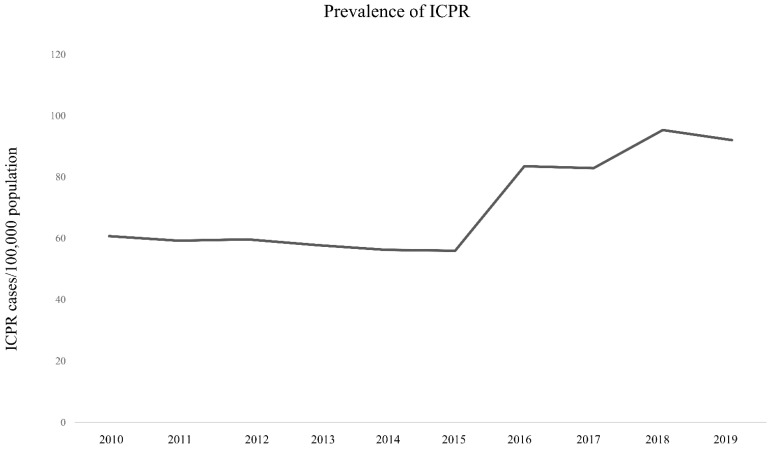
Prevalence of ICPR in South Korea among adult population from 2010 to 2019. ICPR, in-hospital cardiopulmonary resuscitation.

**Figure 2:**
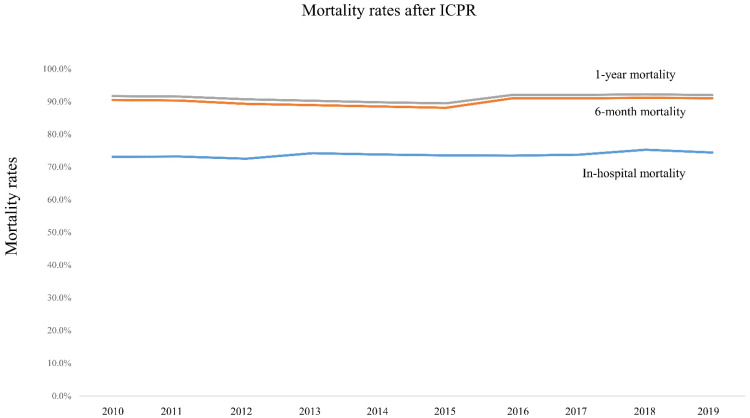
Mortality rates after ICPR from 2010 to 2019. ICPR, in-hospital cardiopulmonary resuscitation.

**Figure 3:**
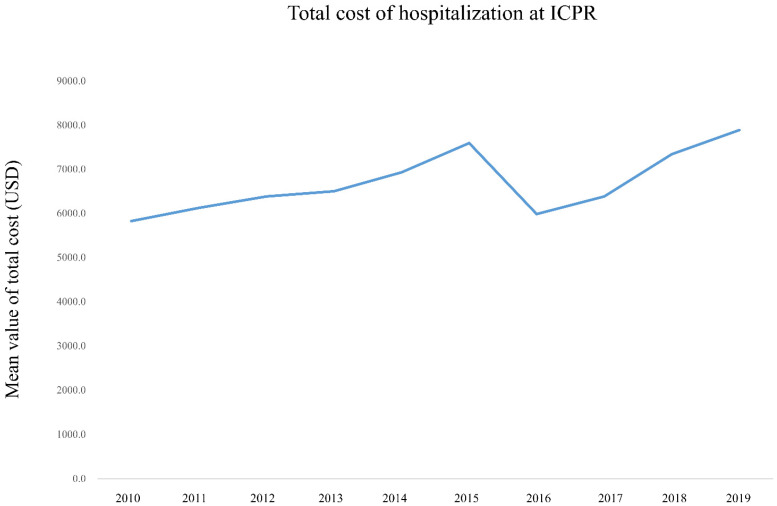
Total cost of hospitalization at ICPR from 2010 to 2019. ICPR, in-hospital cardiopulmonary resuscitation.

Figure 1 and Table S2 show the prevalence of ICPR in South Korea among the adult population from 2010 to 2019. In 2010, 60.7 per 100,000 adults experienced ICPR, and a similar rate was observed until 2015. However, the rate increased to 83.5 per 100,000 adults in 2016 and gradually increased to 92.1 per 100,000 adults in 2019. Figure 2 and Table S3 show mortality rates after ICPR from 2010 to 2019. In-hospital, 6-month, and 1-year mortality rates were 73.1% (17,894/24,486), 90.5% (22,160/24,486), and 91.7% (22,449/24,486), respectively, in 2010. Rates were similar for 10 years through 2019; thus, the in-hospital, 6-month, and 1-year mortality rates were 74.4% (30,080/40,433), 91.0% (36,806/40,433), and 92.0% (37,194/40,433), respectively, in 2019. Figure 3 and Table S4 show the total cost of hospitalization at ICPR from 2010 to 2019. In 2010, the mean value of the total cost of hospitalization was USD 5822.80 (SD: USD 7493.40), which increased to USD 7886.20 (SD: USD 13,071.60) in 2019. 

Figure S2 and Table S5 show the trends in the main diagnosis at ICPR from 2010 to 2019. The proportion of cardiovascular disease as the main diagnosis in 2010 was 29.3% (7174/24,486) and increased to 44.4% (18,472/40,433) in 2019. The proportion of respiratory disease as the main diagnosis in 2010 was 16.2% (3614/24,486) and decreased to 11.2% (4516/40,433) in 2019. The proportion of cancer as the main diagnosis in 2010 was 14.8% (3614/24,486) and decreased to 9.0% (3627/40,433) in 2019. Figure S3 and Table S6 show the trends in the duration of ICPR from 2010 to 2019.

### 3.3. Associated Factors for Discharge Alive

Table 2 shows the results of the multivariable logistic regression model for live discharge after ICPR. Older age (aOR: 0.98, 95% CI: 0.98, 0.99; *p* < 0.001) and residency in rural areas (vs. urban areas) (aOR, 0.91; 95% CI: 0.90, 0.93; *p* < 0.001) were associated with lower odds of live discharge. Compared with cardiovascular disease as the main diagnosis, respiratory disease (aOR: 0.72, 95% CI: 0.70, 0.74; *p* < 0.001), cancer (aOR: 0.38, 95% CI: 0.37, 0.40; *p* < 0.001), and other main diagnoses (aOR, 0.70; 95% CI, 0.68, 0.71; *p* < 0.001) were associated with lower odds of live discharge. In addition, compared with <15 min duration of ICPR, 15–30 min (aOR: 0.42, 95% CI: 0.41, 0.43; *p* < 0.001), 30–45 min (aOR: 0.29, 95% CI: 0.28, 0.30; *p* < 0.001), 45–60 min (aOR: 0.26, 95% CI: 0.24, 0.26; *p* < 0.001), and >60 min (aOR: 0.25, 95% CI: 0.24, 0.26; *p* < 0.001) were associated with lower odds of live discharge.

Compared with unemployed patients, employed patients (aOR: 1.05, 95% CI: 1.03, 1.07; *p* < 0.001) had higher odds of live discharge. Compared with the first quartile (Q1) of household income level, Q2 (aOR: 1.06, 95% CI: 1.03, 1.12; *p* < 0.001), Q3 (aOR: 1.09, 95% CI: 1.06, 1.12; *p* < 0.001), and Q4 (aOR: 1.10, 95% CI: 1.07, 1.12; *p* < 0.001) were associated with higher odds of live discharge.

## 4. Discussion

In this population-based cohort study in South Korea, the prevalence of ICPR increased from 2010 to 2019, but mortality rates remained high during this period: over 90% for 6-month and 1-year mortality after ICPR. Moreover, the financial burden of hospitalization at ICPR increased during the study period. As the main diagnosis at ICPR, cardiovascular increased, while cancer and respiratory disease decreased during the study period. We also show that there were many factors associated with live discharge after ICPR. This study reports important information regarding recent trends in ICPR using real-world data from the NHIS database of South Korea.

In the United States, the prevalence of ICPR among elderly patients was reported as 2.73 events per 1000 admissions from 1992 to 2005 [8]. In South Korea, the prevalence of ICPR was 0.92 events per 1000 admissions in 2019, which was lower than that reported in a previous study [8]. The difference might be due to the inclusion of all adult patients in our study. For non-elderly patients in the United States, the prevalence of ICPR increased from 1.81 per 1000 admissions in 2007 to 2.37 per 1000 admissions in 2012 [5], suggesting that the prevalence of ICPR was relatively low in South Korea. Another cohort study in China reported that the prevalence of ICPR was 4.7 cases per 1000 admissions between 1 January and 31 December 2014, in 12 Beijing hospitals [9], and 1.6 cases per 1000 admissions from 1 April 2011 to 31 March 2013 in the United Kingdom [10]. Therefore, our study shows that the overall prevalence of ICPR among adult patients in South Korea was lower than that in other countries [5,8,9,10].

Our results demonstrated that the frequency of ICPR due to IHCA increased abruptly in 2016 and continuously increased until 2019 in South Korea. Some factors explain this jump. First, many hospitals have been built in South Korea as a result of national planning (https://data.worldbank.org/indicator/SH.MED.BEDS.ZS?locations=KR, accessed on 7 September 2021), which could be associated with the increase in ICPR in South Korea. Second, the super-aged population in South Korea may increase hospitalization rates of elderly patients who were at higher risk of ICPR due to IHCA [11].

A previous meta-analysis in 1998 reported that the rate of survival to discharge after ICPR was 13.4% [12], which was lower than the one in our study (26.2%). However, more recent findings indicate that the rate of survival to discharge after ICPR was 18.4% from 1 April 2011 to 31 March 2013 in the United Kingdom [10], and 34.0% from 1 January 2015 to 31 December 2018 in the United States [13]. In addition to these findings [10,13], we showed that, in recent studies, rates of live discharge following ICPR were higher than those in previous studies [12]. The survival rates after ICPR were 9.8% at 6 months and 8.7% at 1 year in South Korea. A meta-analysis of 40 studies in 2018 reported that the pooled 1-year survival after ICPR following IHCA was 13.4% [4], which was higher than that in our study. As shown in Figure 2, despite some advances in critical care, the mortality rates remain high at over 90% at 6 months and 1 year after ICPR in South Korea.

The financial burden for patients who received ICPR after IHCA increased from 2011 to 2019 in South Korea. The health care cost following ICPR also increased steadily in the United States [14]. In South Korea, the mean total cost of hospitalization at ICPR was USD 6753.50 (SD: USD 10,295.10), and approximately 90% of the total cost was covered by the public health insurance program. This is because the NHIS in South Korea covers 95% of the total medical expenses for treatment and examinations for patients diagnosed with cancer, heart disease, cerebrovascular disease, rare intractable diseases, and severe burns [15].

As our study analyzed a large sample using a nationwide database, there were many interesting factors associated with live discharge after ICPR. As socioeconomic-status-related factors, employment status, urban residency, and higher household income level were associated in this study with higher odds of live discharge. A recent review reported that there was no clear relationship between socioeconomic status and outcomes after ICPR [16]. The impact of socioeconomic status on outcomes after ICPR might be influenced by various health care and social welfare systems in countries. Although most studies use insurance status to evaluate income level [16], we used national household income level, which is a strength of this study.

Interestingly, cancer, as the main diagnosis, was also associated with lower odds of live discharge after ICPR. In previous studies, the survival rate after ICPR for patients with cancer has been reported to be extremely poor [17,18]. A recent cohort study reported that, in oncology wards, 37.2% of patients received potentially avoidable CPR, which was defined as ICPR for patients who had no further chemotherapy plans, were in hospice care, or were expected to have worse clinical courses with irreversible prognosis [19]. In our cohort study, there may have been cases of potentially avoidable ICPR for patients with cancer, which may have affected the live discharge rate.

A longer ICPR duration was also associated with lower odds of live discharge. A retrospective single-center cohort study reported that the duration of ICPR was inversely associated with outcomes, and most of the benefits of ICPR could be achieved in the first 15 min [20]. In our study, 43.1% of patients received ICPR for <15 min, while ICPR duration over 30 min showed similar aORs as those for longer durations (0.29 in the 30–45 min group, 0.26 in the 45–60 min group, and 0.25 in the >60 min group) for live discharge.

This study has several limitations. First, we cannot extract data on important outcomes, such as the return of spontaneous circulation after ICPR, because of the lack of ICD-10 codes in the NHIS database. Second, the NHIS database lacks some important information, such as body mass index, alcohol consumption, and smoking status. Third, there may be residual and unmeasured confounders in our survival analysis of patients who received ICPR. Lastly, the results of our study might have limitations regarding generalizability because clinical practice of ICPR may be influenced by different cultures and health care systems in different countries.

## 5. Conclusions

In conclusion, from 2011 to 2019, there was an increase in the prevalence of ICPR and total costs of hospitalization at ICPR among adult patients in South Korea. The mortality rate remained high for ten years, and many factors, such as unemployment, lower household income level, older age, longer duration of ICPR, cancer as the main diagnosis, and urban residency, were associated with lower odds of live discharge after ICPR. This study provides information useful to hospitalized patients and their physicians in deciding whether to choose to be resuscitated individually considering the clinical benefit of ICPR and financial burden.

## Figures and Tables

**Table 1:** Clinicopathological characteristics of the study population.

Variable	Mean (SD) or N (%)
Age	70.0 (15.2)
Sex, male	180,389 (60.4)
Have a job at ICPR	153,053 (51.2)
Residence at ICPR	
Urban area	129,057 (43.2)
Rural area	169,619 (56.8)
Household income level at ICPR	
Q1	93,778 (31.4)
Q2	46,226 (15.5)
Q3	58,628 (19.6)
Q4	94,424 (31.6)
Unknown	5620 (1.9)
Main diagnosis at ICPR	
Cardiovascular disease	115,159 (38.6)
Respiratory disease	41,959 (14.0)
Cancer	33,498 (11.2)
Other	108,060 (36.2)
Admitting department	
IM	166,779 (55.8)
Non-IM	131,897 (44.2)
Duration of ICPR	
<15 min	128,869 (43.1)
15–30	86,534 (29.0)
30–45	40,848 (13.7)
45–60	19,227 (6.4)
>60 min	16,402 (5.5)
LOS at ICPR	9.9 (13.1)
Total cost for hospitalization at ICPR, USD	6753.5 (10,295.1)
Insurance coverage	6030.2 (9490.5)
CCI	6.0 (3.9)
Type of hospital	
Tertiary general hospital	112,290 (37.6)
General hospital	153,320 (51.3)
Other hospital	33,066 (11.1)
Total hospital bed number	
<1000	253,184 (84.8)
≥1000	45,492 (15.2)
Result of treatment	
Discharge and same hospital follow up	25,617 (8.6)
Transfer to long-term facility care center	8396 (2.8)
Death within hospitalization after ICPR	220,493 (73.8)
Discharge and other outpatient clinic follow-up	44,170 (14.8)
Year of ICPR	
2010	24,486 (8.2)
2011	24,169 (8.1)
2012	24,606 (8.2)
2013	24,092 (8.1)
2014	23,696 (7.9)
2015	23,811 (8.0)
2016	35,880 (12.0)
2017	35,894 (12.0)
2018	41,609 (13.9)
2019	40,433 (13.5)

SD, standard deviation; ICPR, in-hospital cardiopulmonary resuscitation; IM, internal medicine; USD, United States Dollar; LOS, length of hospital stays; CCI, Charlson comorbidity index.

**Table 2:** Multivariable logistic regression model for live discharge after ICPR.

Variable	aOR (95% CI)	*p*-Value
Age	0.98 (0.98, 0.99)	<0.001
Sex, male	0.99 (0.98, 1.01)	0.431
Have a job at ICPR	1.05 (1.03, 1.07)	<0.001
Residence at ICPR		
Urban area	1	
Rural area	0.91 (0.90, 0.93)	<0.001
Household income level at ICPR		
Q1	1	
Q2	1.06 (1.03, 1.09)	<0.001
Q3	1.09 (1.06, 1.12)	<0.001
Q4	1.10 (1.07, 1.12)	<0.001
Unknown	1.08 (1.01, 1.15)	0.019
Main diagnosis at ICPR		
Cardiovascular disease	1	
Respiratory disease	0.72 (0.70, 0.74)	<0.001
Cancer	0.38 (0.37, 0.40)	<0.001
Other	0.70 (0.68, 0.71)	<0.001
Admitting department		
IM	1	
Non-IM	1.03 (1.02, 1.05)	<0.001
Duration of ICPR		
<15 min	1	
15–30	0.42 (0.41, 0.43)	<0.001
30–45	0.29 (0.28, 0.30)	<0.001
45–60	0.26, 0.25, 0.27)	<0.001
>60	0.25 (0.24, 0.26)	<0.001
CCI, point	1.03 (1.02, 1.03)	<0.001
Type of hospital		
Tertiary general hospital	1	
General hospital	0.83 (0.81, 0.84)	<0.001
Other hospital	1.06 (1.02, 1.09)	0.001
Total hospital bed number		
<1000	1	
≥1000	0.90 (0.88, 0.93)	<0.001
Year of ICPR		
2010	1	
2011	1.00 (0.96, 1.05)	0.921
2012	1.01 (0.97, 1.056)	0.605
2013	0.89 (0.85, 0.93)	<0.001
2014	0.90 (0.86, 0.94)	<0.001
2015	0.91 (0.87, 0.95)	<0.001
2016	0.92 (0.89, 0.96)	<0.001
2017	0.89 (0.86, 0.93)	<0.001
2018	0.85 (0.81, 0.88)	<0.001
2019	0.88 (0.85, 0.92)	<0.001

aOR, adjusted odds ratio; CI, confidence interval; ICPR, in-hospital cardiopulmonary resuscitation; IM, internal medicine; CCI, Charlson comorbidity index.

## Data Availability

All data will be available upon reasonable request to corresponding author.

## Supplementary Materials

The following supporting information can be downloaded at: https://www.mdpi.com/article/10.3390/jpm12030377/s1, Table S1. The ICD-10 codes used by comorbidity to compute the Charlson comorbidity index, Table S2. Prevalence of ICPR in South Korea among the adult population from 2010 to 2019, Table S3. Mortality rates after ICPR from 2010 to 2019, Table S4. Mean value of total cost of hospitalization at ICPR from 2010 to 2019 in USD, Table S5. Trends in the main diagnosis at ICPR from 2010 to 2019, Table S6. Trends in the duration of ICPR from 2010 to 2019, Figure S1. Trends in the main diagnosis at ICPR from 2010 to 2019, Figure S2. Trends in the duration of ICPR from 2010 to 2019, Figure S3. Flow chart depicting study population selection process.
