# Supplementary material for: Trends in In-Hospital Cardiopulmonary Resuscitation from 2010 through 2019: A Nationwide Cohort Study in South Korea
Source: J Pers Med. 2022 Mar 1;12(3):377. doi: 10.3390/jpm12030377 (PMC8954519; doi:10.3390/jpm12030377)
Supplement: Supplementary file 1 [file jpm-12-00377-s001.zip › jpm-1585019-supplementary/Table S2.pdf]

Table S2. Prevalence of ICPR in South Korea among the adult population from 2010 to 2019.

Year	2010	2011	2012	2013	2014	2015	2016	2017	2018	2019
ICPR cases	24486	24169	24606	24092	23696	23811	35880	35894	41609	40433
Population	40,359,211	40,813,272	41,256,396	41,709,764	42,141,075	42,567,533	42,960,165	43,298,097	43,649,724	43,920,954
Prevalence per 10,0000	60.7	59.2	59.6	57.8	56.2	55.9	83.5	82.9	95.3	92.1

ICPR, In-hospital cardiopulmonary resuscitation
