# Supplementary material for: Trends in In-Hospital Cardiopulmonary Resuscitation from 2010 through 2019: A Nationwide Cohort Study in South Korea
Source: J Pers Med. 2022 Mar 1;12(3):377. doi: 10.3390/jpm12030377 (PMC8954519; doi:10.3390/jpm12030377)
Supplement: Supplementary file 1 [file jpm-12-00377-s001.zip › jpm-1585019-supplementary/Table S3.pdf]

Table S3. Mortality rates after ICPR from 2010 to 2019

	2010	2011	2012	2013	2014	2015	2016	2017	2018	2019
Hospital mortality	73.1%	73.2%	72.5%	74.2%	73.8%	73.5%	73.5%	73.7%	75.2%	74.4%
6-month mortality	90.5%	90.3%	89.3%	88.9%	88.5%	88.1%	91.0%	91.0%	91.1%	91.0%
1-year mortality	91.7%	91.5%	90.7%	90.3%	89.8%	89.5%	92.0%	92.0%	92.2%	92.0%

ICPR, in-hospital cardiopulmonary resuscitation
