# Supplementary material for: Trends in In-Hospital Cardiopulmonary Resuscitation from 2010 through 2019: A Nationwide Cohort Study in South Korea
Source: J Pers Med. 2022 Mar 1;12(3):377. doi: 10.3390/jpm12030377 (PMC8954519; doi:10.3390/jpm12030377)
Supplement: Supplementary file 1 [file jpm-12-00377-s001.zip › jpm-1585019-supplementary/Table S4.pdf]

Table S4. Mean value of total cost of hospitalization at ICPR from 2010 to 2019 by USD

	2010	2011	2012	2013	2014	2015	2016	2017	2018	2019
Mean value of total cost	5822.8	6123.2	6381.8	6499.8	6929.1	7592.5	5982.7	6380.6	7339.7	7886.2
SD	7493.4	8061.7	8334.5	8513.5	8978.9	10078.5	10014.1	11160.8	11810.1	13071.6

ICPR, in-hospital cardiopulmonary resuscitation; USD, United states dollars; SD, standard deviation
