# Supplementary material for: Trends in In-Hospital Cardiopulmonary Resuscitation from 2010 through 2019: A Nationwide Cohort Study in South Korea
Source: J Pers Med. 2022 Mar 1;12(3):377. doi: 10.3390/jpm12030377 (PMC8954519; doi:10.3390/jpm12030377)
Supplement: Supplementary file 1 [file jpm-12-00377-s001.zip › jpm-1585019-supplementary/Table S5.pdf]

Table S5. Main diagnosis at ICPR from 2010 to 2019

	2010	2011	2012	2013	2014	2015	2016	2017	2018	2019
Cardiovascular disease	29.3%	29.3%	30.6%	31.5%	32.2%	32.7%	45.2%	46.1%	44.4%	47.3%
Respiratory disease	16.2%	16.3%	17.1%	16.6%	16.2%	16.8%	11.5%	11.2%	12.9%	11.2%
Cancer	14.8%	14.5%	13.6%	12.7%	12.8%	12.3%	9.2%	8.9%	9.3%	9.0%
Other	39.8%	39.8%	38.8%	39.1%	38.9%	38.3%	34.1%	33.8%	33.4%	32.5%

ICPR, in-hospital cardiopulmonary resuscitation
