# Supplementary material for: Trends in In-Hospital Cardiopulmonary Resuscitation from 2010 through 2019: A Nationwide Cohort Study in South Korea
Source: J Pers Med. 2022 Mar 1;12(3):377. doi: 10.3390/jpm12030377 (PMC8954519; doi:10.3390/jpm12030377)
Supplement: Supplementary file 1 [file jpm-12-00377-s001.zip › jpm-1585019-supplementary/Table S6.pdf]

Table S6. Trends in the duration of ICPR from 2010 to 2019

	2010	2011	2012	2013	2014	2015	2016	2017	2018	2019
<15 min	41.7%	42.2%	44.1%	46.0%	47.2%	48.1%	43.3%	44.0%	43.2%	41.7%
15-30	28.0%	28.5%	27.7%	27.6%	26.3%	25.6%	31.8%	31.7%	31.8%	32.1%
30-45	14.3%	14.3%	13.7%	13.1%	13.5%	13.4%	14.4%	13.7%	14.5%	14.4%
45-60	8.0%	7.9%	7.7%	7.3%	7.0%	6.8%	6.0%	5.8%	5.9%	5.5%
>60 min	8.0%	7.1%	6.8%	6.1%	6.1%	6.2%	4.5%	4.8%	4.6%	4.4%

ICPR, in-hospital cardiopulmonary resuscitation
