# Supplementary figures and images for: Trends in In-Hospital Cardiopulmonary Resuscitation from 2010 through 2019: A Nationwide Cohort Study in South Korea
Source: J Pers Med. 2022 Mar 1;12(3):377. doi: 10.3390/jpm12030377 (PMC8954519; doi:10.3390/jpm12030377)

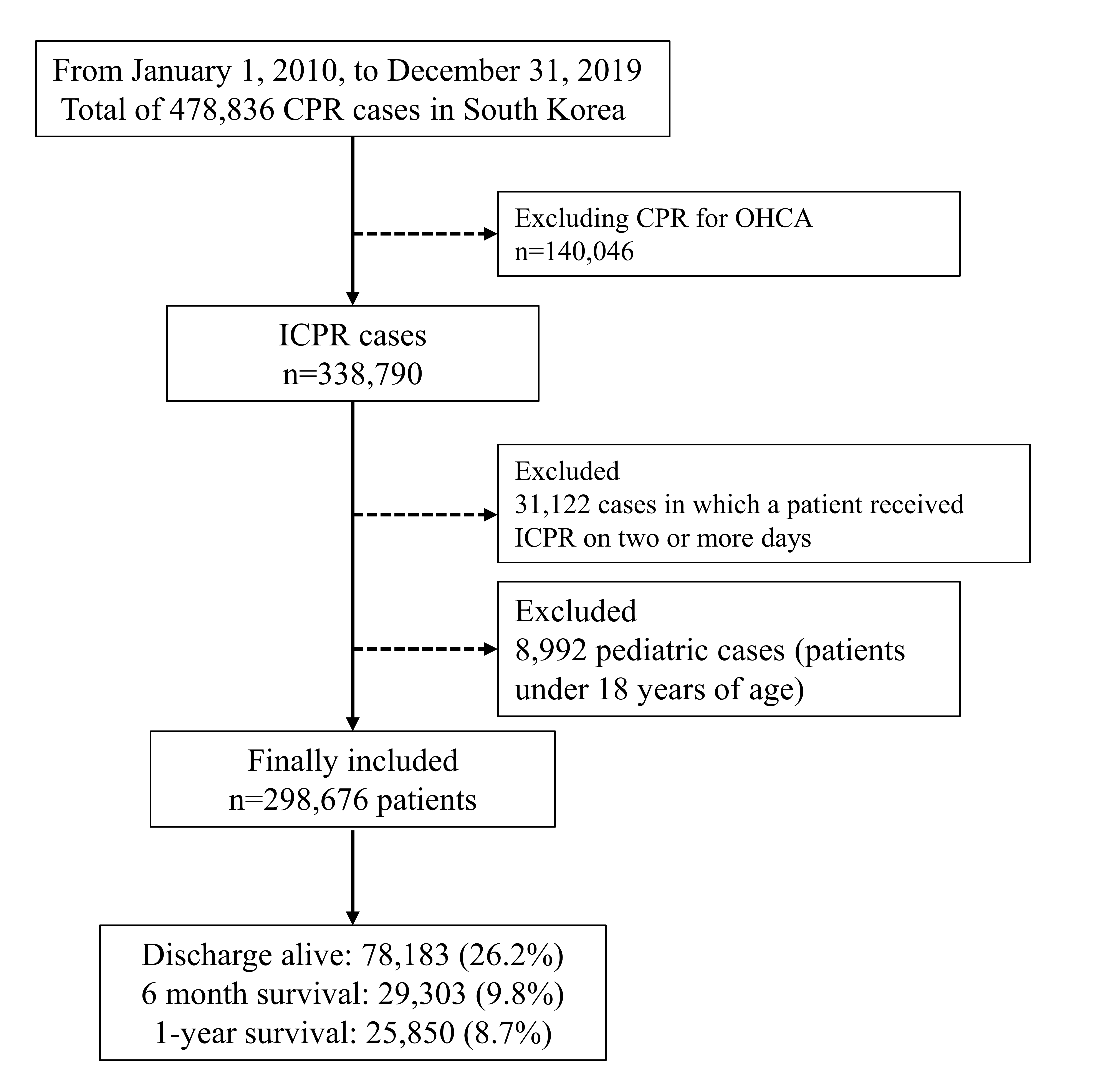

Supplement: Supplementary file 1 [file jpm-12-00377-s001.zip › jpm-1585019-supplementary/Figure S1_sub_rev.tif]

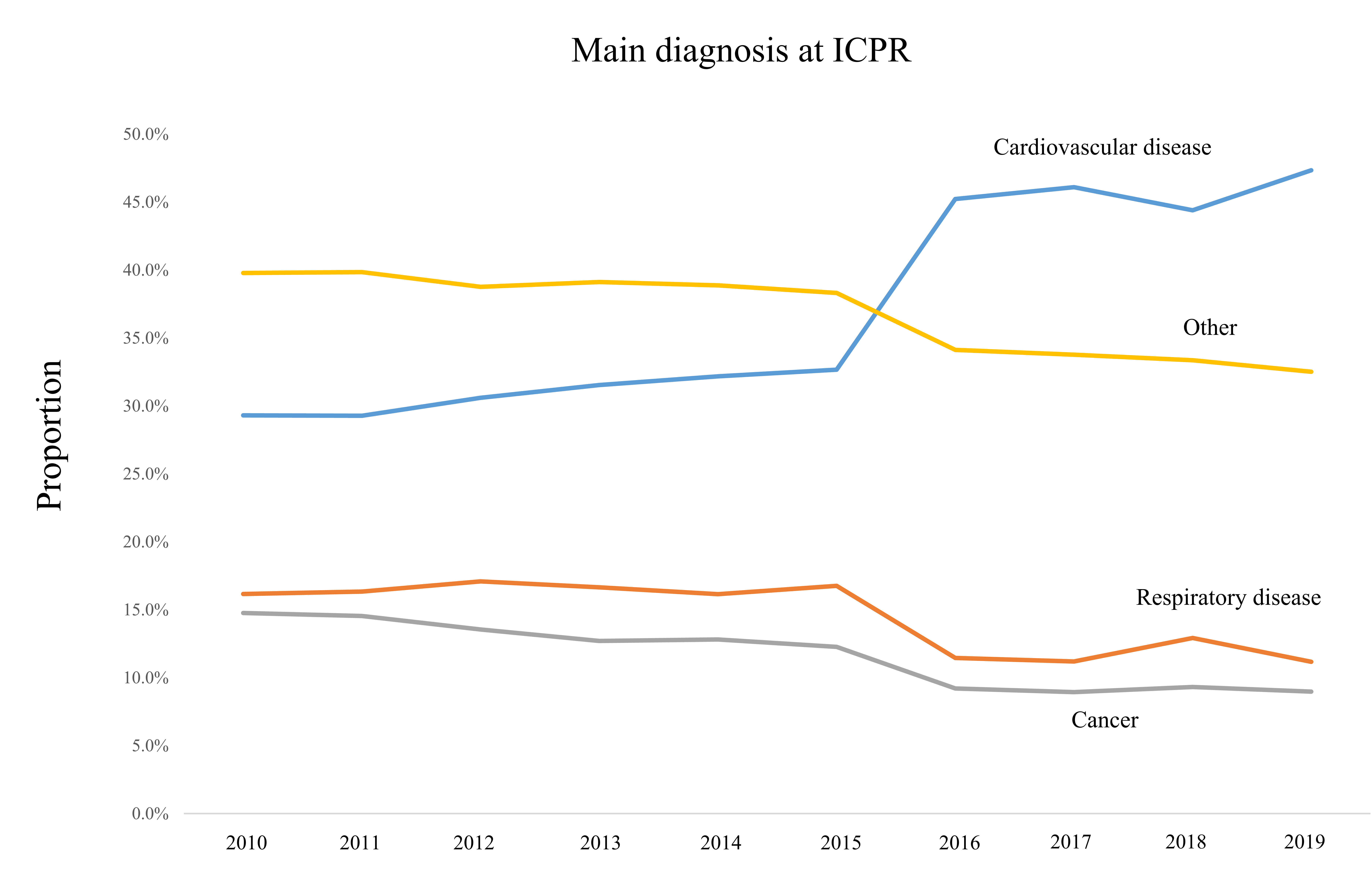

Supplement: Supplementary file 1 [file jpm-12-00377-s001.zip › jpm-1585019-supplementary/Figure S2_Sub_rev.tif]

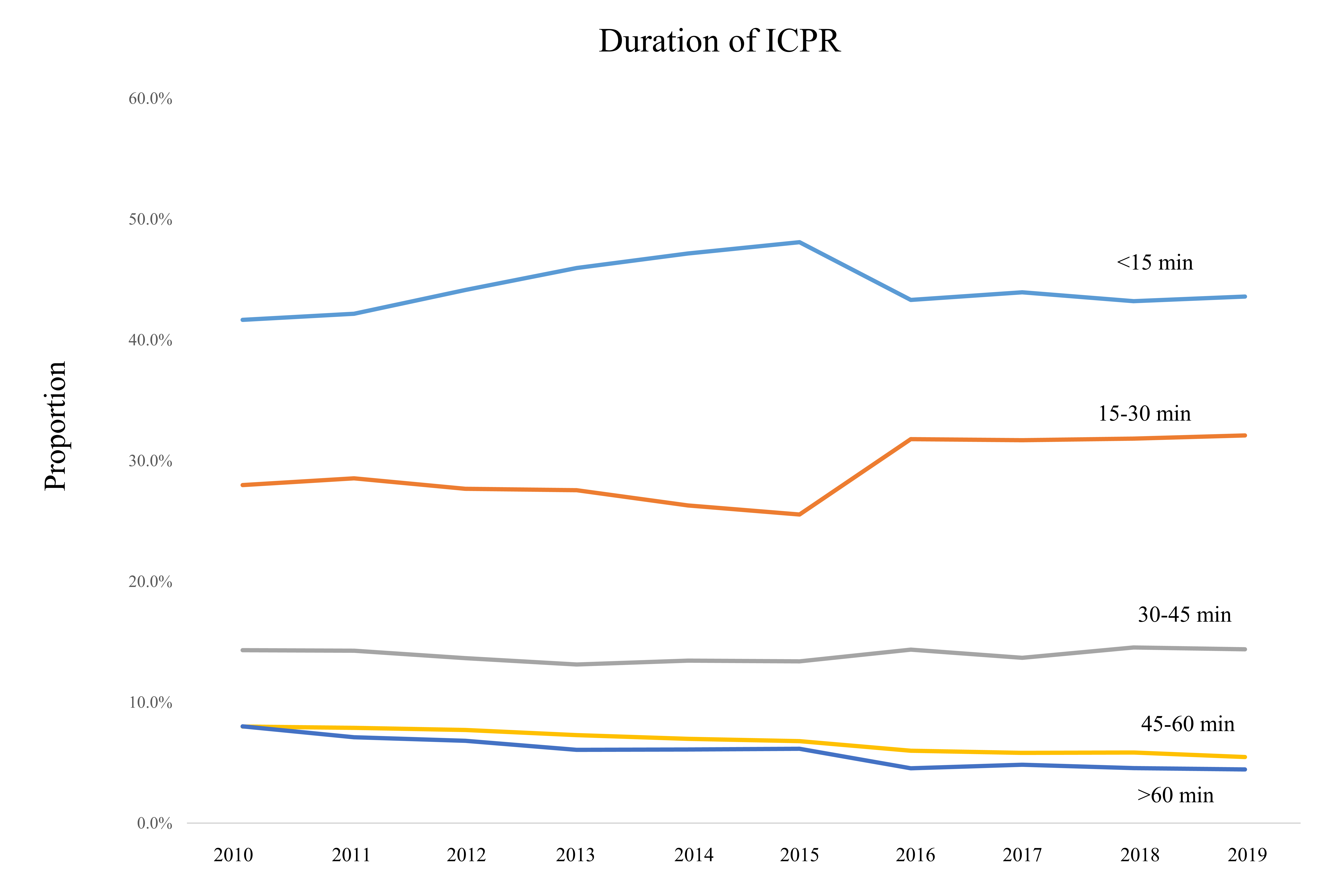

Supplement: Supplementary file 1 [file jpm-12-00377-s001.zip › jpm-1585019-supplementary/Figure S3_sub_rev.tif]
